# Fusion or Fission: The Destiny of Mitochondria In Traumatic Brain Injury of Different Severities

**DOI:** 10.1038/s41598-017-09587-2

**Published:** 2017-08-23

**Authors:** Valentina Di Pietro, Giacomo Lazzarino, Angela Maria Amorini, Stefano Signoretti, Lisa J. Hill, Edoardo Porto, Barbara Tavazzi, Giuseppe Lazzarino, Antonio Belli

**Affiliations:** 1 Neuroscience and Ophthalmology Research Group, Institute of Inflammation and Ageing, School of Clinical and Experimental Medicine, College of Medical and Dental Sciences, University of Birmingham, Edgbaston, B15 2TT Birmingham, UK; 2 National Institute for Health Research Surgical Reconstruction and Microbiology Research Centre, Queen Elizabeth Hospital, Edgbaston, B15 2TH Birmingham, UK; 3 Institute of Biochemistry and Clinical Biochemistry, Catholic University of Rome, Largo F. Vito 1, 00168 Rome, Italy; 4 Division of Neurosurgery, Department of Neurosciences Head and Neck Surgery, S. Camillo Hospital, Circonvallazione Gianicolense 87, 00152 Rome, Italy; 5 Department of Biomedical and Biotechnological Sciences, Division of Medical Biochemistry, University of Catania, Viale A. Doria 6, 95125 Catania, Italy

## Abstract

Mitochondrial dynamics are regulated by a complex system of proteins representing the mitochondrial quality control (MQC). MQC balances antagonistic forces of fusion and fission determining mitochondrial and cell fates. In several neurological disorders, dysfunctional mitochondria show significant changes in gene and protein expression of the MQC and contribute to the pathophysiological mechanisms of cell damage. In this study, we evaluated the main gene and protein expression involved in the MQC in rats receiving traumatic brain injury (TBI) of different severities. At 6, 24, 48 and 120 hours after mild TBI (mTBI) or severe TBI (sTBI), gene and protein expressions of fusion and fission were measured in brain tissue homogenates. Compared to intact brain controls, results showed that genes and proteins inducing fusion or fission were upregulated and downregulated, respectively, in mTBI, but downregulated and upregulated, respectively, in sTBI. In particular, OPA1, regulating inner membrane dynamics, cristae remodelling, oxidative phosphorylation, was post-translationally cleaved generating differential amounts of long and short OPA1 in mTBI and sTBI. Corroborated by data referring to citrate synthase, these results confirm the transitory (mTBI) or permanent (sTBI) mitochondrial dysfunction, enhancing MQC importance to maintain cell functions and indicating in OPA1 an attractive potential therapeutic target for TBI.

## Introduction

The crucial role of mitochondria to maintain cell life has become more evident over the last few decades. Physiologically, these organelles are involved in many key cellular processes including being responsible for the supply of energy (which is produced by the electron transport chain coupled to oxidative phosphorylation and ATP production), to act as an effective storage for calcium ions and to actively participate in intracellular trafficking^1^. Pathologically, mitochondria are involved in the generation of the so-called oxidative/nitrosative stress, caused by excess production of reactive oxygen and nitrogen species (ROS and RNS, respectively), as well as in the induction of apoptosis^2, 3^.

The shift of normal mitochondrial activity to pathological activity lies in an initial functional-to-dysfunctional transition state causing, in turn, the activation of dangerous pathological reactions, potentially leading to definitive, irreversible mitochondrial damage. Reverting mitochondrial malfunctioning may be crucial in avoiding irreversible processes being triggered, such as apoptosis, thus perventing the physiology-to-pathology shift. Either physiologically or pathologically, the life of mitochondria is maintained by a complex network of proteins, interconnected by their activity and regulated by complex post-translational modifications involved in the control of mitochondrial fission, fusion and autophagy^4, 5^. The continuous process of fission and fusion is part of the usual mitochondrial network dynamics, causing macroscopic changes to the organelle morphology, and is considered to be the mitochondrial quality control (MQC) system for eukaryotic cells^6^.

Under physiological conditions, occasionally dysfunctional mitochondria are fused with other healthy mitochondria with the aim to restore normal mitochondrial function. Persistently dysfunctional mitochondria are promptly separated from functional mitochondria by fission and they will be removed and recycled via mitochondrial autophagy^7^. Under pathological conditions, when any type of physical-chemical stimulus is operating, fusion is activated in order to protect mitochondria whilst verifying that they are still functional. Prolonged pathological stressors inhibit fusion, stimulate fission and cause mitochondrial fragmentation, with consequent triggering of apoptosis and inevitable cell death^6–9^.

Many conserved GTPase proteins are involved in mitochondrial fusion and fission dynamics, among these are mitofusins (MFN1 and MFN2) and dominant optic atrophy 1 (OPA1) which are required for the fusion of mitochondrial outer (OM) and inner membranes (IM), respectively^10^. Dynamin-related protein 1 (DRP1) and mitochondrial Fission 1 protein (FIS1) are the main mitochondrial fission mediators^11^. The dynamic interactions among these proteins and the role of OPA1 proteolytic post-processing in balancing fusion and fission are summarised in the Supplementary Table 1 and Fig. S1.

In particular, it has been shown that OPA1 deficiency leads to loss of mitochondrial fusion, disorganization of cristae membranes^12^, severely reduced phosphorylating capacity^13^ and higher sensitivity to apoptosis^7–9, 12–14^. On the other hand, overexpression of OPA1 does protect against apoptosis associated with neurodegeneration^15^, working synergistically with Presenilin-associated Rhomboid-like protein (PARL)^16^, specifically involved in the regulation of OPA1 oligomerization^17^.

In total, eight isoforms of OPA1 are expressed in different tissues^18^, each of them encodes an OPA1 precursor that is imported into the mitochondrion, where the N-terminal mitochondrial targeting sequence is removed to produce a long isoform of OPA1 (L-OPA1) ultimately embedded in the IM. In normal physiological conditions, about half of OPA1 exists as L-OPA1. Generally, L-OPA1 contains a S1 cleavage site, but OPA1 splice-forms 4, 6, 7 and 8 can also contain an additional S2 protease cleavage site^7, 8, 10, 11^. The cleavage at both sites creates short forms (S-OPA1) that are no longer anchored to the membrane^10, 11^.

Mitochondrial fusion depends on the presence of both L- and S-OPA1 which assemble into heterodimeric complexes enabling inner membrane fusion and cristae organization^19, 20^. However, the L-OPA1 isoform alone is sufficient for stress-induced mitochondrial fusion^20^. Various stress conditions including apoptotic stimulation, low ATP level or dissipation of mitochondrial membrane potential, trigger the complete conversion of L-OPA1 into S-OPA1, which forms multimeric S-OPA1 complexes inhibiting mitochondrial fusion and promoting mitochondrial outer membrane permeabilisation^21^. The persistence of these stressors leads to prevention of fusion and to the stimulation of fission proteins (DRP1), thereby causing: i) the increase in number of damaged mitochondria; ii) the release of apoptotic signals (cytochrome c); iii) the removal of permanently dysfunctional mitochondria through mitophagy with the involvement of PINK1 and PARK2^7, 8, 17, 21^.

Many different proteases intervene in OPA1 processing, either directly or indirectly. Among the most important, OMA1 cleaves all splice-forms of L-OPA1 at the S1 site after decreases in mitochondrial membrane potential^21^. The i-AAA protease YME1 catalyses the proteolytic cleavage at the S2 site (therefore generating S-OPA1 only from a subset of OPA1 isoforms) and is required for adjustment in mitochondrial fusion by properly balancing the L-OPA1/S-OPA1 ratio^21–24^.

It is well known that dysfunctional mitochondria are a common feature not only of chronic neurodegenerative diseases such as multiple sclerosis^25^, Alzheimer’s disease^26^, amyotrophic lateral sclerosis^27^, Parkinson’s disease^28^, but also of acute neurodegeneration caused by cerebral ischemia^29^ and traumatic brain injury (TBI)^30–32^. TBI is a major health and socioeconomic problem throughout the world in adults below 40 years of age. TBI is a complicated pathological process consisting of a primary insult (the impact force acting on the brain tissue) directly inducing a scarcely predictable secondary insult characterized by a cascade of biochemical, metabolic and molecular changes causing profound mitochondrial malfunctioning in cerebral cells^30–32^. The magnitude and duration of such changes strictly depend on the severity of the injury^31, 33, 34^. The imbalance between a higher energy demand for restoring cell homeostasis and the decreased ATP production from dysfunctional mitochondria provoke a well defined cell energy crisis^31, 33^. However, cellular responses after mild TBI (mTBI) or after severe TBI (sTBI) are very often reflected by opposite molecular strategies, leading to upregulation or downregulation of specific pathways in mTBI and vice-versa in sTBI^34, 35^. However, notwithstanding the abundant literature, to date little is known about the changes in gene and protein expression involved in the MQC following TBI^36^.

In the present study, we analysed gene and protein expressions of the main molecules involved in mitochondrial fusion and fission in cohorts of control, mildly- and severely-injured rats with the aim to investigate any correlation between mitochondrial dynamics and TBI severity.

## Results

### Expression of genes regulating mitochondrial fusion after graded TBI

**Figure 1:**
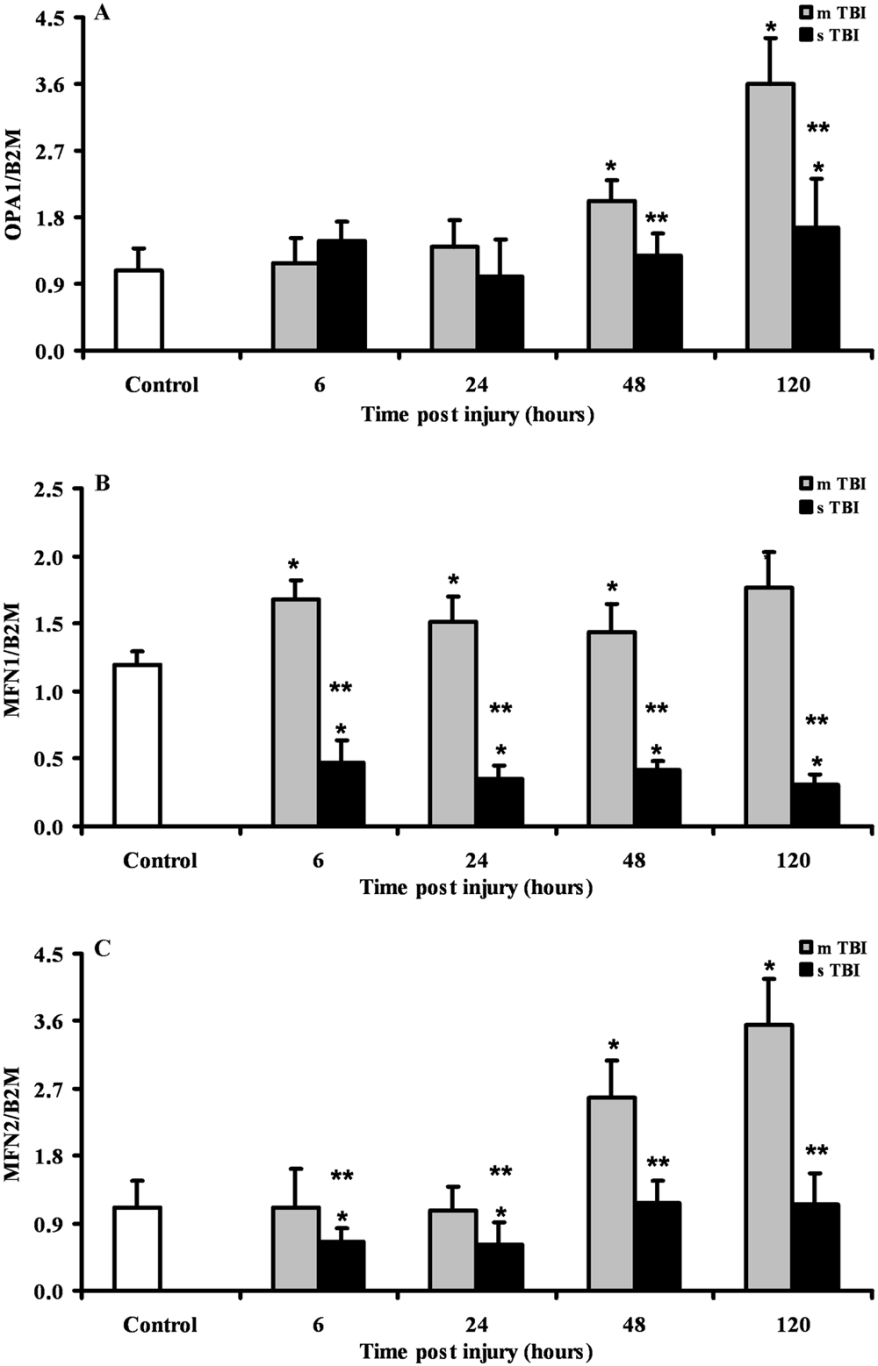
Changes in the expression of fusion-related genes (OPA1, MFN1 and MFN2) in brain tissue homogenates of rats receiving different severities of TBI (mTBI and sTBI) at various times post-impact. Values are the mean of 6 animals. Standard deviations are represented by vertical bars. Gene expressions were calculated relatively to the housekeeping gene β-2-microglobulin (B2M). *Significantly different from controls, p < 0.05. **Significantly different from the corresponding time of mTBI rats, p < 0.05.

Figure 1 reports changes in the expressions of OPA1, MFN1 and MFN2, in rats receiving mTBI or sTBI at various times after injury. A significant increase in these genes regulating fusion were recorded in mTBI rats, particularly at 48 and 120 hours post-injury. With respect to controls, OPA1 increased by 1.9 and 3.3 times (p < 0.01), MFN1 increased by 1.2 and 1.4 times (p < 0.05) and MFN2 increased by 2.3 and 3.2 times (p < 0.05), respectively. Rats experiencing sTBI showed no changes in OPA1, a dramatic downregulation of MFN1 anytime post-injury (p < 0.05 compared to both controls and corresponding times of mTBI animals) and an early downregulation with subsequent normalization of MFN1.

**Figure 2:**
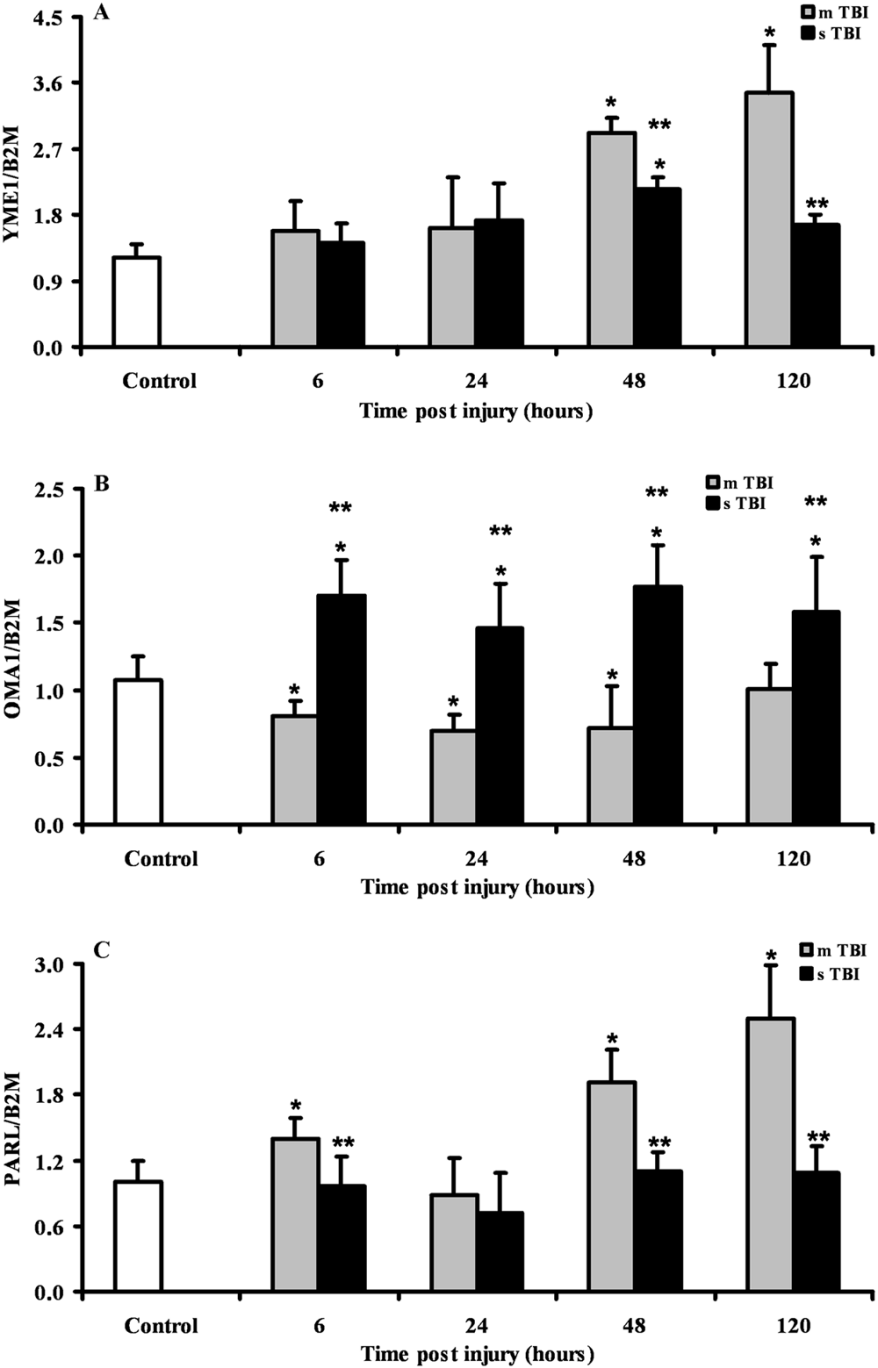
Changes in the expression of genes regulating the post-translational processing of OPA1 (YME1, OMA1 and PARL) in brain tissue homogenates of rats receiving TBI of graded severity (mTBI and sTBI) at different times post-impact. Values are the mean of 6 animals. Standard deviations are represented by vertical bars. Gene expressions were calculated relatively to the housekeeping gene β-2-microglobulin (B2M). *Significantly different from controls, p < 0.05. **Significantly different from the corresponding time of mTBI rats, p < 0.05.

Since mitochondrial fusion is strictly regulated by the proteolytic scission and oligomerization of OPA1, we measured the expressions of the genes YME1, OMA1 and PARL, all of which encode for the most important proteins involved in OPA1 post-translational processing. Data reported in Fig. 2 show that after mTBI, YME1 increased by 2.5 and 3 times (p < 0.05 compared to controls) at 48 and 120 hours, respectively. Differently, OMA1 was downregulated at 6, 24 and 48 hours (p < 0.05 compared to controls) and returned to the value recorded in controls at 120 hours post-injury. The gene expression of PARL after mTBI was similar to that of YME1, with significant increases by 1.9 and 2.5 times over the value of controls at 48 and 120 hours post injury (p < 0.05). In sTBI rats, YME1 was modestly upregulated only at 48 hours post-trauma (p < 0.05 compared to controls), whilst OMA 1 was constantly upregulated anytime post-injury (p < 0.05 compared to both controls and mTBI rats) by approximately 1.6 times. No significant changes in PARL expression were detected any time after sTBI respect to controls, even though PARL in sTBI-injured rats was lower at 6, 48 and 120 hours post impact than values measured at corresponding times in mTBI rats (p < 0.05).

### Expression of genes regulating mitochondrial fission after graded TBI

**Figure 3:**
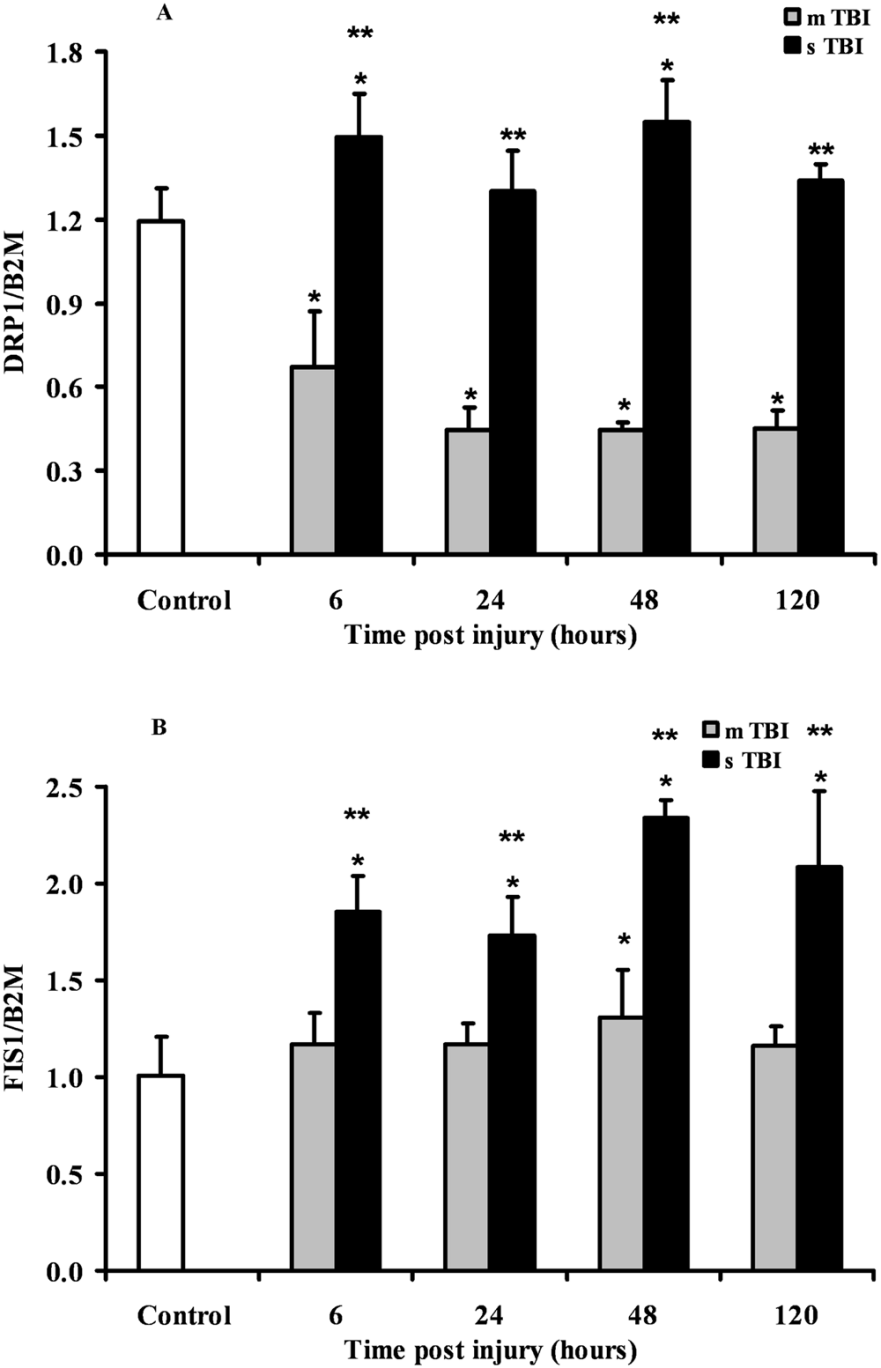
Changes in the expression of fission-related genes (DRP1 and FIS1) in brain tissue homogenates of rats receiving TBI of graded severity (mTBI and sTBI) at different times post-impact. Values are the mean of 6 animals. Standard deviations are represented by vertical bars. Gene expressions were calculated relatively to the housekeeping gene β-2-microglobulin (B2M). *Significantly different from controls, p < 0.05. **Significantly different from the corresponding time of mTBI rats, p < 0.05.

The time course changes in the expression of the genes controlling mitochondrial fission following graded TBI (DRP1 and FIS1), are illustrated in Fig. 3. In mTBI rats, a constant downregulation of DRP1 by approximately 35% of the value of controls was recorded at any time point after injury (p < 0.05). In these animals, no significant changes occurred to the gene expression of FIS1. The expression of DRP1 in severely injured rats was significantly higher than the values recorded in controls at 6 and 48 hours (p < 0.05), but more than double the values found in mTBI rats anytime post-impact (p < 0.05). The gene expression of FIS1 was significantly higher anytime post-injury than values determined in controls (p < 0.05) and mTBI rats (p < 0.05).

### Expression of genes regulating mitophagy after graded TBI

**Figure 4:**
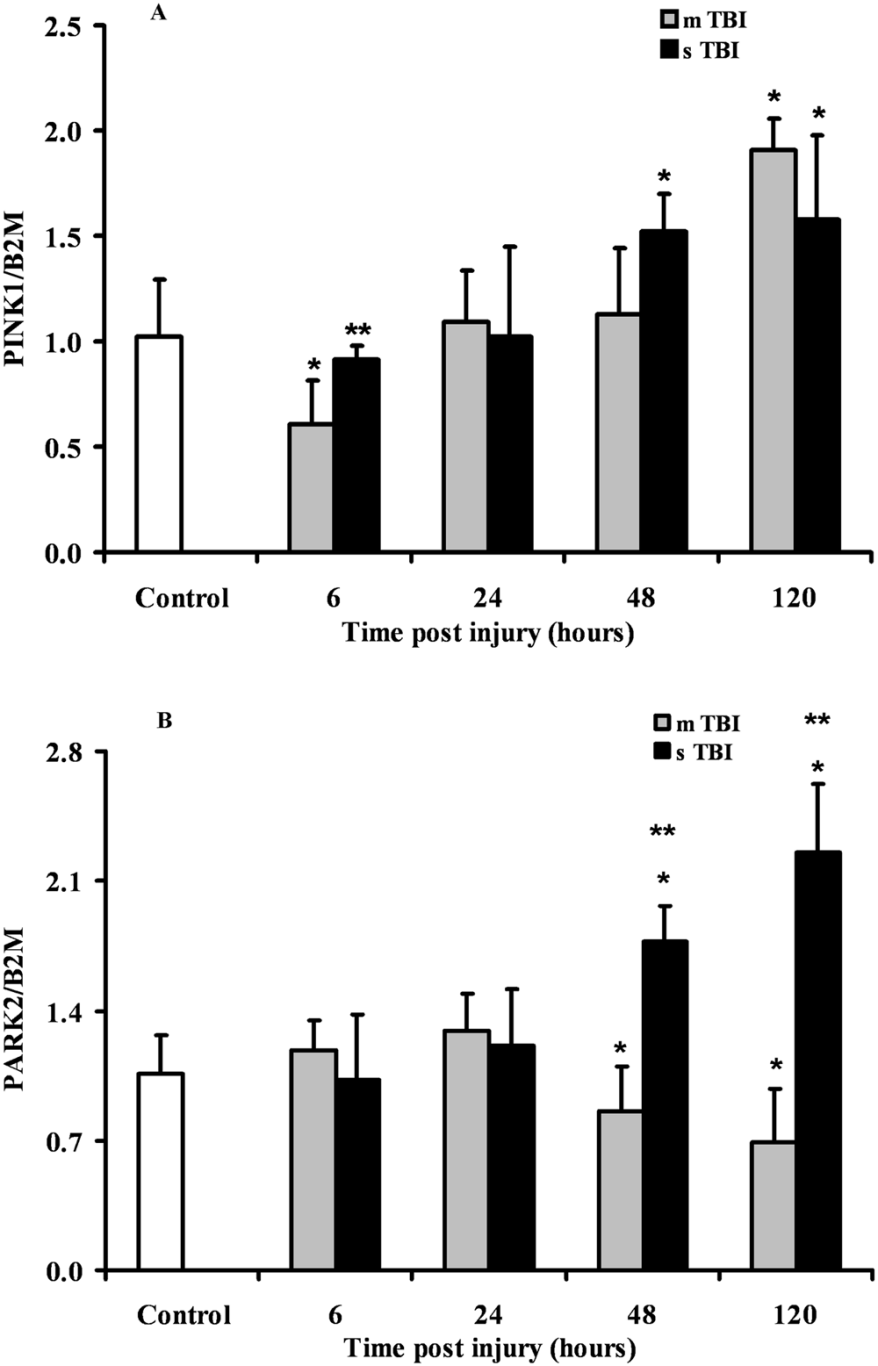
Changes in the expression of mitophagy-related genes (PINK1 and PARK2) in brain tissue homogenates of rats receiving TBI of graded severity (mTBI and sTBI) at different times post-impact. Values are the mean of 6 animals. Standard deviations are represented by vertical bars. Gene expressions were calculated relatively to the housekeeping gene β-2-microglobulin (B2M). *Significantly different from controls, p < 0.05. **Significantly different from the corresponding time of mTBI rats, p < 0.05.

The two genes PINK1 and PARK2 encoding for the proteins playing crucial roles in mitophagy regulation were differently affected by mTBI or sTBI (Fig. 4). After mTBI, PINK1 showed an early (6 hours post-injury) downregulation (about 45% decrease with respect to the value of controls, p < 0.05), followed by normalization at 24 and 48 hours and later increases of up to 190% compared to sham-operated rats (p < 0.05). The expression of PARK2 did not vary at 6 and 24 hours, but decreased by 20% and 35% at 48 and 120 hours, respectively (p < 0.05 compared to controls).

In sTBI rats, PINK1 was not significantly different from controls at 6 and 24 hours post-impact and was overexpressed by 1.5 and 1.6 times at 48 and 120 hours post-injury, respectively (p < 0.05 respect to controls). Similarly, PARK2 expression did not vary at 6 and 24 hours but was significantly upregulated by approximately 1.7 and 2.1 times at 48 and 120 hours, respectively (p < 0.05 compared to both controls and mTBI rats).

### Fusion and fission proteins and mitochondrial mass quantification after graded TBI

**Figure 5:**
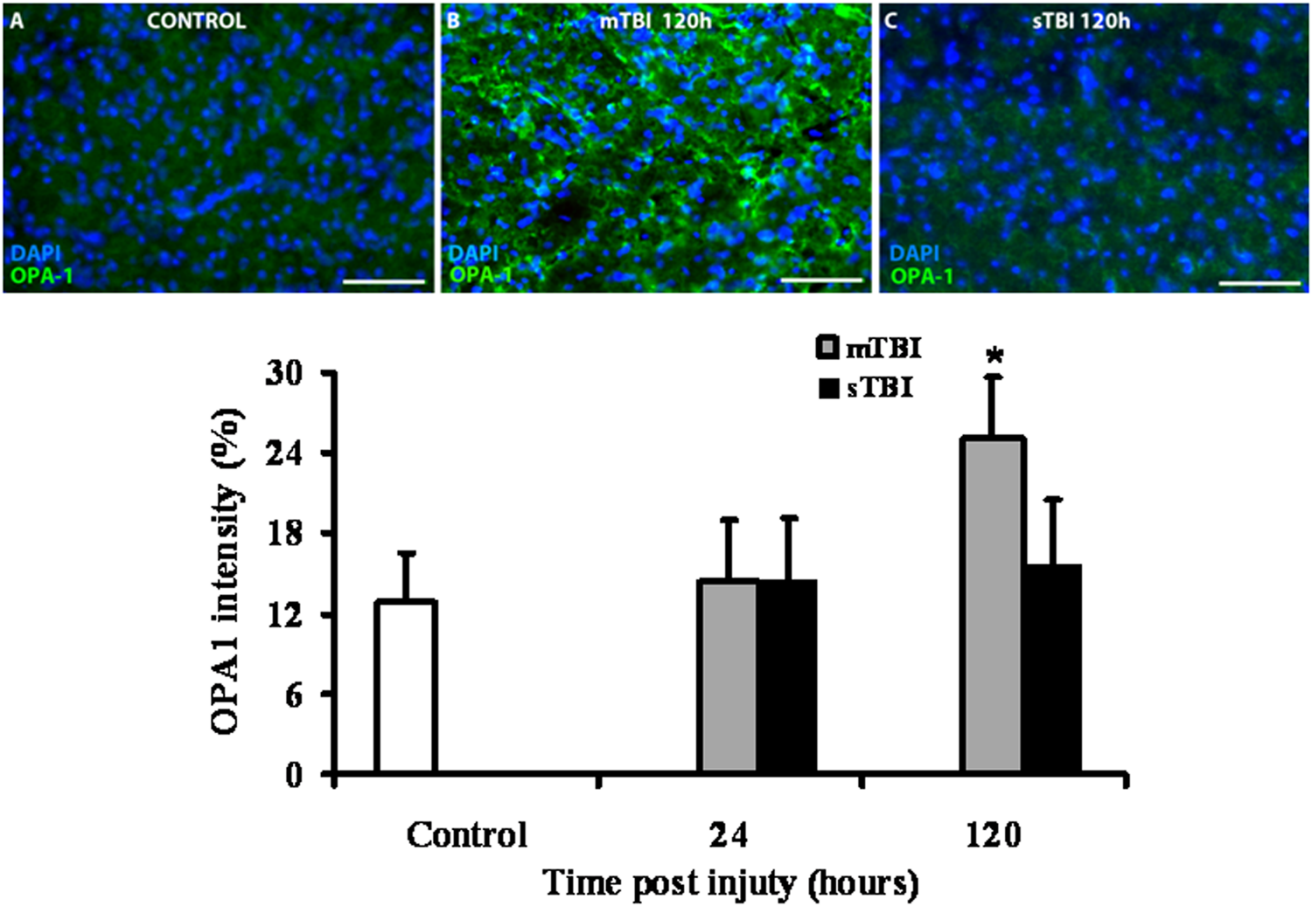
Immuno-staining with anti-OPA1 antibody of brain tissue of rats receiving TBI of graded severity (mTBI and sTBI) at 24 and 120 hours post-impact. In Panels A, B and C, representative staining of control, mTBI and sTBI rats, respectively, are illustrated. In the bar graph, mean values of immuno-staining with anti-OPA1 antibody are shown. Values of histograms are the mean of 6 animals. Standard deviations are represented by vertical bars. *Significantly different from controls, p < 0.05.

From the genes involved in mitochondrial fusion and fission, we selected OPA1, OMA1 and DRP1 to evaluate the expression of the corresponding proteins as a function of injury severity. As illustrated in Fig. 5, immunohistochemistry revealed no differences in cerebral OPA1 of rats subjected to either mTBI or sTBI, at 24 hours post-injury. Conversely, the immunoreactive OPA1 clearly showed that brains of mTBI rats had double levels of OPA1 at 120 hours post-impact compared to both control and sTBI animals (p < 0.05).

**Figure 6:**
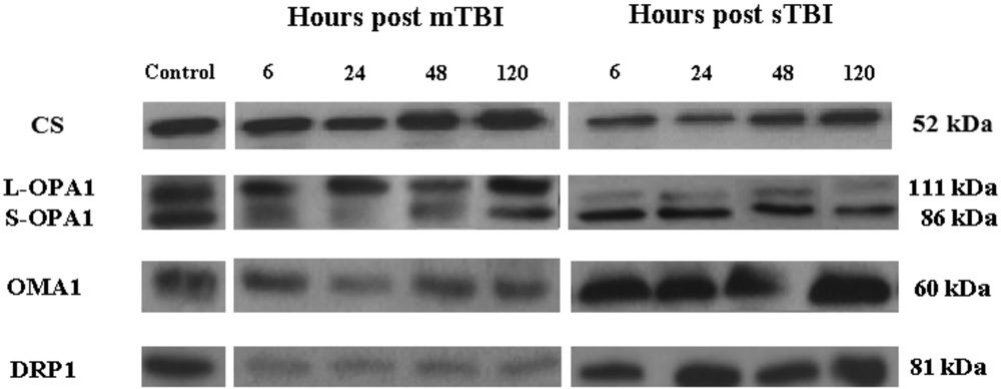
Representative Western blot analysis of the expression of citrate synthase (CS) as a measure of mitochondrial mass, and of proteins involved in fusion (L-OPA1, S-OPA1, OMA1) and fission (DRP1). The analysis was carried out in brain tissue homogenates of rats receiving TBI of graded severity (mTBI and sTBI) at different times post-impact.

**Figure 7:**
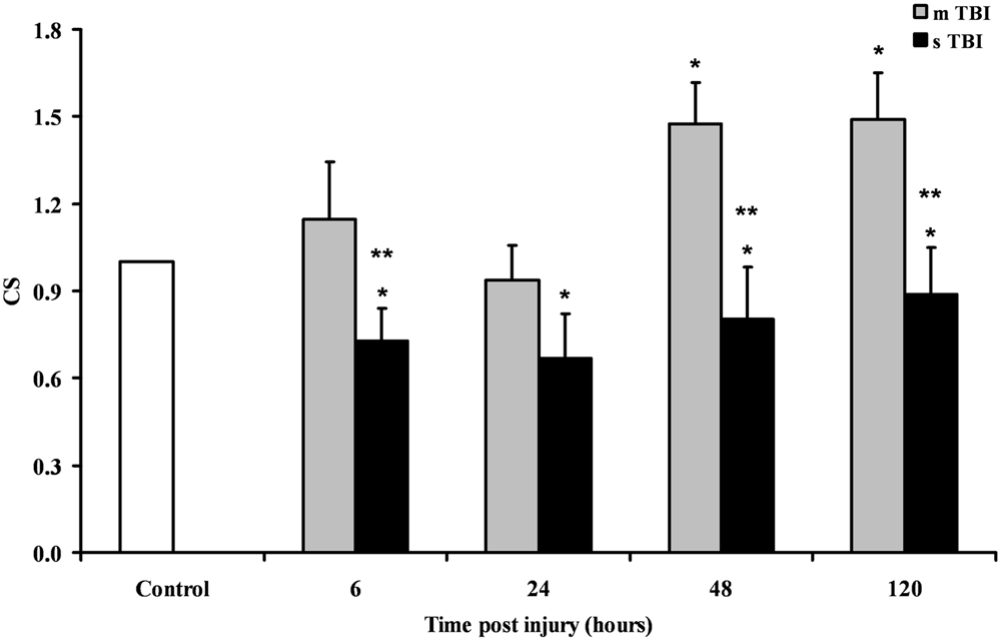
Changes in CS (as a mitochondrial mass indicator) in brain tissue homogenates of rats receiving TBI of graded severity (mTBI and sTBI) at different times post-impact. Values are the mean of 6 animals. Standard deviations are represented by vertical bars. *Significantly different from controls, p < 0.05. **Significantly different from the corresponding time of mTBI rats, p < 0.05.

**Figure 8:**
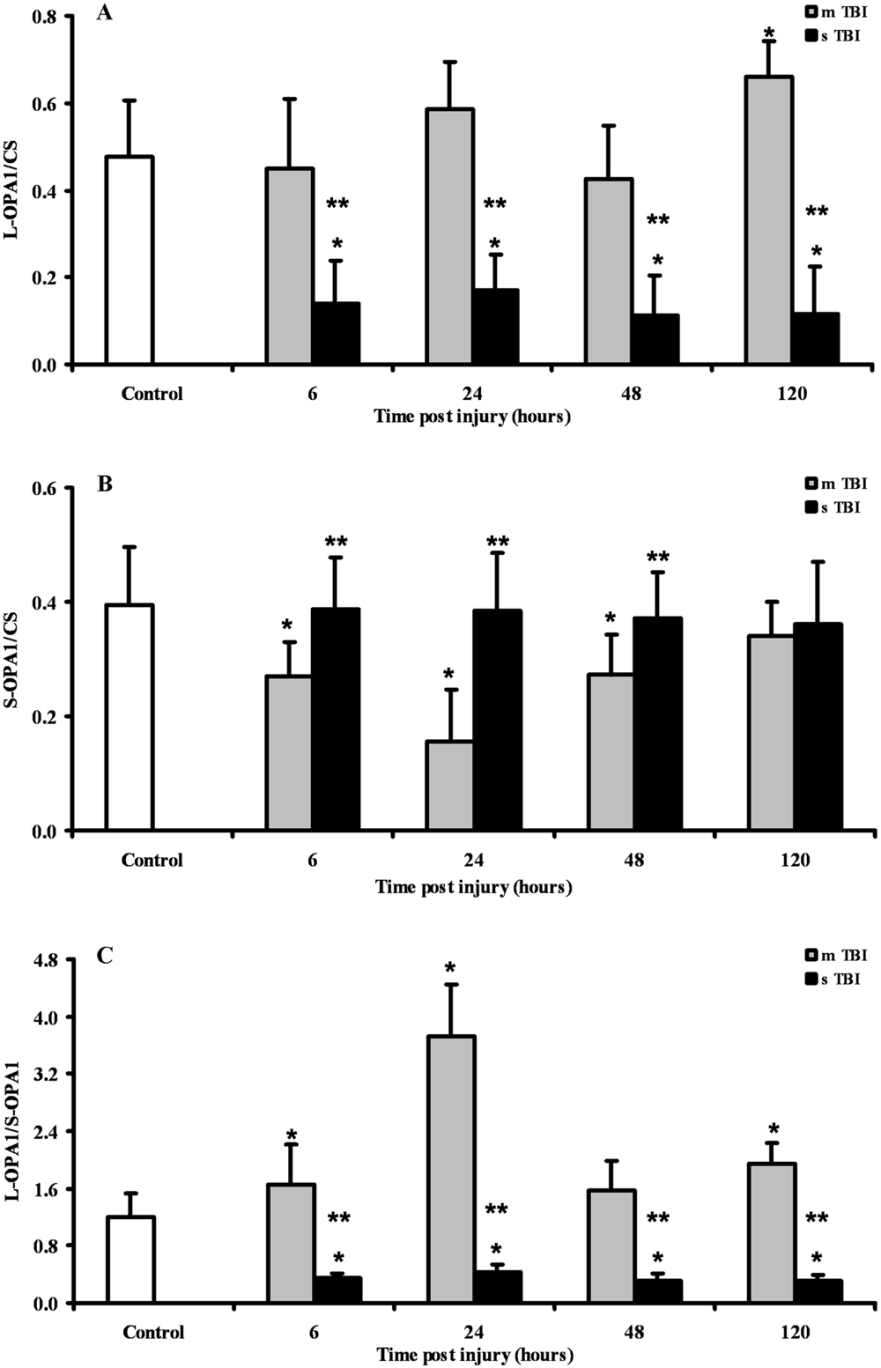
Semi-quantitative determination of the proteolytic products of OPA1 (L-OPA1 and S-OPA1) in brain tissue homogenates of rats receiving TBI of graded severity (mTBI and sTBI) at different times post-impact. The L-OPA1/S-OPA1 ratio is also reported. Values are the mean of 6 animals. Standard deviations are represented by vertical bars. Protein expressions were calculated relatively to CS (as a mitochondrial mass indicator). *Significantly different from controls, p < 0.05. **Significantly different from the corresponding time of mTBI rats, p < 0.05.

**Figure 9:**
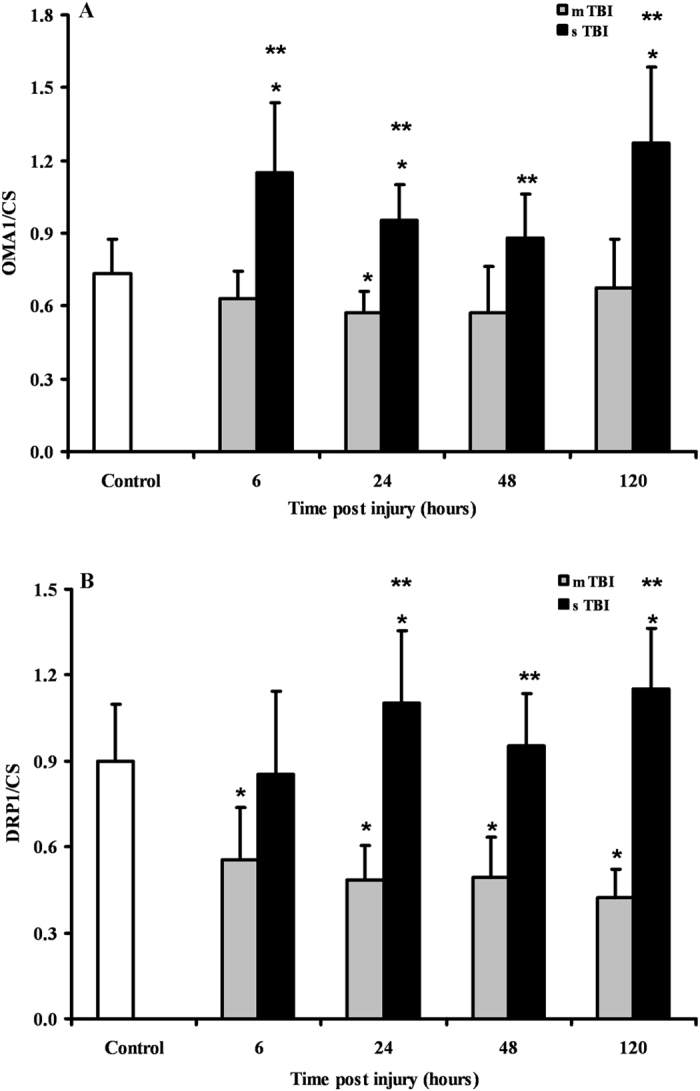
Semi-quantitative determination of OMA1 and DRP1 in brain tissue homogenates of rats receiving TBI of graded severity (mTBI and sTBI) at different times post-impact. Values are the mean of 6 animals. Standard deviations are represented by vertical bars. Protein expressions were calculated relatively to CS (as a mitochondrial mass indicator). *Significantly different from controls, p < 0.05. **Significantly different from the corresponding time of mTBI rats, p < 0.05.

Since immunohistochemistry could not distinguish between the two cleaved forms of the OPA1 protein, we performed a Western blot analysis aimed to allow semi-quantitative determination of L-OPA1 and S-OPA1, as well as of the protease OMA1 and of the main fission protein DRP1, in brain tissue of controls, and of mTBI and sTBI rats at different times after injury. In order to obtain a fairly reasonable indication of changes in mitochondrial numbers in the injured animals, we also carried out the immunoblot analysis of citrate synthase (CS), which is considered a good indicator of the mitochondrial mass^37, 38^. A representative gel indicating the bands corresponding to these proteins is shown in Fig. 6. The densitometric quantification of CS, illustrated in Fig. 7, indicates that mTBI rats had no changes in mitochondrial mass at 6 and 24 hours and a significant increase at 48 and 120 hours post-injury (+45%, p < 0.05 compared to controls). Conversely, CS in sTBI animals underwent significant decrease at all time points post-injury (p < 0.05 compared to both controls and mTBI rats). This was particularly evident early on after injury. Figure 8 shows that L-OPA1 in mTBI rats remained unchanged at 6, 24 and 48 hours but increased significantly by 40% at 120 hours post injury (p < 0.05 compared to controls). However, L-OPA1 in the sTBI rats was significantly decreased at all time points post-injury (p < 0.05 compared to both controls and mTBI rats). Concomitantly, S-OPA1 was significantly lowered at 6, 24 and 48 hours following mTBI (p < 0.05 compared to controls) and did not differ from controls at any time point after sTBI (p < 0.05 compared to mTBI rats). Consequently, from the differential post-translational processing of OPA1, the L-OPA1/S-OPA1 ratio was strikingly different after mTBI compared to sTBI. As reported in Fig. 9, in brain tissue exposed to mTBI the L-OPA1/S-OPA1 ratio significantly increased post-injury (p < 0.05 compared to controls). Conversely, the L-OPA1/S-OPA1 ratio in brain tissue exposed to sTBI was approximately 30% of control values (p < 0.05 compared to both sham and mTBI injured animals). In accordance with these data, the semi-quantitation of OMA1 (Fig. 9) demonstrated a decrease in mildly injured animals that only reached significance by 24 hours post-injury (p < 0.05 compared to controls) compared to the significant increases at all time points in severely injured rats (up to double of the value of controls; p < 0.05 compared to both controls and mTBI rats). Protein expression of DRP1 in mTBI rats (Fig. 9) was less than half the value found in controls, at each time point after impact (p < 0.05). DRP1 expression after sTBI was 40% higher compared to controls at 24 and 120 hours (p < 0.05) and also significantly higher than the corresponding values of mTBI at any time point post-injury (p < 0.05).

A schematic summary of the changes in genes and proteins expressions of the MQC after graded TBI is shown in Supplementary Tables 2 and 3.

## Discussion

Mitochondrial dysfunction in cerebral tissue has widely been addressed as a central phenomenon of the post-traumatic neurometabolic cascade occurring after TBI^39–41^. Notwithstanding the deep interest towards mitochondria, there is little understanding about mitochondrial dynamics after TBI^36^.

Results of the present study provide, for the first time, a satisfactory vision of the (dys)regulation of the main components supervising fusion, fission and mitophagy following graded TBI. According to the data obtained in our trauma model, which mostly produces diffuse axonal injury^42, 43^, it is possible to postulate that cerebral cells after mTBI are committed to increase fusion and to concomitantly decrease fission and mitophagy. Conversely, brain cells after sTBI are more prone to activate fission and mitophagy, the two phenomena being paralleled by a remarkable downregulation of fusion.

Rats experiencing mTBI showed an upregulation of the genes encoding for OPA1 and MFN2, the two main proteins activating fusion of the IM and OM^10, 21^, respectively, which was similar either for magnitude or for timing post impact. Since both proteins are needed for fusion completion^10, 21^, our results suggest that under these conditions the process of mitochondrial tethering and OM fusion could successfully occur thanks to increases in MFN2 and giving the concomitant increase in OPA1, IM membrane fusion could also lead to termination of the fusion process. It should, however, be noted that an YME1-mediated post-translational proteolytic OPA1 cleavage is necessary to generate more L-OPA1^21–24^, which is the pro-fusion, anti-apoptotic form of this protein^21^. A significant inhibition of the OMA1-mediated conversion of L-OPA1 into S-OPA1, which is the anti-fusion, pro-apoptotic form of this protein^7, 10, 21^, is also required. According to our immunohistochemical data, OPA1 protein was homogeneously overexpressed in all brain areas at 120 hours only, after mTBI. Additionally, brain cells showed an overexpression and a downregulation of the genes encoding for YME1 and OMA1, respectively, suggesting a possible prevalence of L-OPA1. Western blot analysis clearly showed that in mTBI rats a remarkable predominance of the protective L-OPA1 form was observed early post injury, and almost equal levels of L-OPA1 and S-OPA1 were found at longer times. It is worth underlining that no change (early post-injury) or increase (at 48 and 120 hours post-impact) in protein expression of CS paralleled the overexpression of OPA1 and YME1 genes, together with the increase in L-OPA1 protein expression and the decrease in gene and protein expressions of OMA1. Altogether, these findings strongly suggest a commitment of mitochondria towards fusion rather than fission following mTBI, which resulted in a net increase in cerebral mitochondrial mass at later time points post-injury (Fig. 7). This should not only induce the prevention of apoptosis^12, 44^, but also mitochondrial cristae remodelling^12, 13, 45^ with improvement of ETC coupled to OXPHOS^46^.

Activation of mitochondrial fusion following mTBI was accompanied by a significant inhibition of fission. The downregulation of the DRP1 gene (by almost 3 times) should have had remarkable beneficial effects since it has been demonstrated that increase of the mitochondrial division inhibitor-1 (Mdivi-1) of DRP1, by decreasing oligomeric assembly and GTP binding affinity of DRP1^47^, reduces cell death in different cellular and animal models^47–50^. Moreover, it has very recently been shown that mitochondrial fission inhibition by Mdivi-1 reduces cortical cell loss and improves spatial memory after TBI in mice^51^. The sum of fusion activation and fission inhibition in our mTBI animals has had the cumulative effect to produce no activation of mitophagy, as indicated by the unchanged (or modest change of) expressions of the genes encoding for PINK1 and PARK2, i.e. the two genes regulating the PINK1/PARK2 pathway of mitophagy^21, 52^. The slight increase of PINK1, only late post-injury, might be related to the additional PINK1 biochemical activities rather than activator of mitophagy, including regulator of complex I function and promoter of cell survival via interaction of calcium homeostasis^53^. Together with the data on CS, this corroborates the indication that mTBI induces only a permanent increase in the number of dysfunctional mitochondria.

A very different pattern was observed in rats receiving sTBI. No change in OPA1 (both of gene and protein expressions), dramatic downregulation of MFN1 throughout the post-injury period (6-120 hours), and of MFN2 in the early phases post-impact (6 and 24 hours), produced an overall inhibition of the mitochondrial fusion process. Additionally, since a concomitant overexpression of YME1 and OMA1 occurred, cerebral cells of sTBI animals clearly showed an extensive OPA1 proteolytic cleavage that almost exclusively generated S-OPA1 (Fig. 8). This is a potent stimulus towards the formation of S-OPA1 complexes capable of causing mitochondrial OM permeabilisation and release of cytochrome c, one of the most powerful signals of the intrinsic pathway of apoptosis^54^.

Concomitantly to the increase in the pro-apoptotic S-OPA1 signal, sTBI injured brains stimulated fission by simultaneously overexpressing both genes encoding for the two fission proteins, DRP1 and FIS1. This is particularly relevant since fission is more efficient when both proteins are present, and importantly noting that DRP1 is anchored at the mitochondrial OM through binding to receptor-like molecule, by FIS1^21, 55^. In sTBI rats, inhibition of fusion, generation of the pro-apoptotic S-OPA1 and activation of fission had the inevitable effect of activating mitophagy, as indicated by the significant overexpression of the genes encoding for PINK1 and PARK2. Hence, after sTBI an increased number of permanently dysfunctional mitochondria are present, necessitating a prompt removal from the cellular environment via the activation of the PINK1/PARK2 pathway regulating mitophagy^52, 53^. This indication is clearly supported by the concomitant overexpression of both PINK1 and PARK2 genes (Fig. 4), as well as a mean 20% decrease in CS and reduction in brain mitochondrial mass after sTBI (Fig. 7).

The data presented here were obtained from the same cohorts of control, mTBI and sTBI animals in which we found that mTBI causes a transient mitochondrial malfunctioning characterized by alteration of the mitochondrial phosphorylating capacity^56, 57^, a transitory imbalance in the cell antioxidant defences^58^, and, a temporary glucose dysmetabolism^57^. In these experiments, sTBI, produced permanent mitochondrial malfunctioning as demonstrated by loss of their phosphorylating capacity and profound ATP depletion^56, 57^, permanent decrease in the main low molecular weight antioxidant GSH^58^, permanent glucose dysmetabolism characterized by marked hyperglycolysis^57^ and continued excitotoxicity caused by increase of glutamate and other excitatory amino acids, as well as a decrease of N-acetylaspartate^56, 59^.

Putting together these previous findings with those referring to mitochondrial dynamics reported in the present study, it is possible to depict a complex cascade of molecular events which are triggered by the traumatic insult and involving changes of cellular metabolism and metabolites, of enzymatic activities, of protein and gene expressions. How these modifications will evolve is depending on the TBI severity. All of these changes are deeply interconnected and encompass mitochondria as either main players or as main targets. Both of these mitochondrial roles have serious repercussions on destiny and survival of mitochondria (and cells) and also determine which processes participating to the MQC (fusion, fission, mitophagy) will prevail.

TBI severity dictates whether mitochondrial dysfunction is successfully rescued. In mTBI, the biochemical and molecular strategy avoids mitochondria to work at their maximal capacity despite being dysfunctional and allows high metabolic flow through mitochondria only when the activation of fusion has properly occurred^56, 57^. In sTBI, the permanent mitochondrial dysfunction produces “true” hyperglycolysis and a sustained energy deficit caused by loss of the mitochondrial phosphorylating capacity^56, 57^. Therefore, a vicious cycle is triggered that imbalances mitochondrial dynamics towards the prevalent inhibition of fusion, activation of fission and subsequent mitophagy, provoking irreversible damages to cerebral cells. The slight but significant decreases in mitochondrial mass (Fig. 8), accompanied by prolonged mitochondrial dysfunction, is a valid explanation for the lack of metabolic recovery occurring following sTBI^56–59^.

The processes described here for mTBI indicate a lag time before that fusion prevails over fission and mitophagy. This confirms previous findings^35, 56–59^ and strongly suggests that during this period of time cells must be defined as biochemically, metabolically and genetically vulnerable. A second mild injury occurring within this period might have fatal consequences. Therefore, in the case of concussive brain injuries^60^, the present results might be relevant to explain why repeated concussions taking place during this period of “molecular vulnerability” at least cause a disproportion between the second impact and the time of normalisation of brain metabolism and resolution of symptoms^61^. But, they may also explain why, in a minority of cases, repeated concussions may give raise to the catastrophic second impact syndrome^31, 41, 61^. Even the aetiopathogenesis of CTE caused by recurrent concussions^62–64^ might be connected to continuous mitochondrial malfunctioning and imbalance of the MQC.

In conclusion, we have here shown that the complex systems supervising to mitochondrial dynamics, undergo to significant changes following a traumatic insult. Whether MQC provides recovery of mitochondria (promoting cell survival) through fusion activation and fission inhibition, or elimination of mitochondria (promoting apoptosis and cell death) through fusion inhibition, and fission and mitophagy activation, strictly depends on the force (energy) that acts on the skull and is transferred to the brain tissue (mTBI = recovery; sTBI = no recovery). To our knowledge, only Mdivi-1, the inhibitor of DRP1, has been tested as a potential drug targeting the MQC in head trauma^51^. It is however conceivable, that new drugs targeting the protease OMA1, responsible for the conversion of most of OPA1 into S-OPA1, might have beneficial effects after sTBI^65^.

The main limitation of this and our previous studies is that they are pre-clinical rodent studies. However, since these and previous findings have been determined in the brain tissue of the same cohorts of animals, it is possible to affirm that we produced results connecting glucose dysmetabolism^57^, energy imbalance^56, 57^, oxidative/nitrosative stress^58^, NAA^56, 59^ and amino acid imbalance^59^, with alterations of the MQC. These interconnected biochemical modifications appear to ultimately decide not only the destiny of mitochondria but the destiny of brain recovery following graded TBI. It is evident that increased efforts should be dedicated to develop newer drugs to effectively target mitochondrial functions for the treatment of sTBI.

## Methods

### Experimental protocol

The experimental protocol was approved by the Ethical Committee of the Catholic University of Rome, in accordance with the United States Public Health Service’s Policy on Humane Care and Use of Laboratory Animals. As previously described, male Wistar rats of 300–350 g were randomly divided into the following groups: 1) sham-operated control; 2) mild diffuse TBI (mTBI group); 3) severe diffuse TBI (sTBI group)^56–59^. All animals were anesthetized receiving 35 mg/kg b.w. of ketamine and 0.25 mg/kg b.w. of midazolam by i.p. injection. Since we were interested in a type of trauma producing a diffuse axonal injury, graded TBI (mTBI or sTBI) was induced according to the weight drop impact acceleration model characterized by causing diffuse axonal damage^42, 43^. After anesthesia, a metal disk was fixed onto the central portion of the skull, between the coronal and lambdoid sutures, in order to prevent skull fracture and to homogenously distribute the force acting at the time of impact. Mild or severe TBI were induced by dropping a cumulative weight of 450 g from 1 or 2 m height and knowing to cause, respectively, a mTBI or a sTBI either biochemically or histopathologically^31, 33, 42, 43, 56–59^. To grossly account for the difference in injury severity using this model of TBI, a mortality rate of 11.8% (4/34 rats) was recorded in the group of sTBI, whilst all mTBI rats survived to the impact for the desired time. At 6, 24, 48, and 120 h from injury, rats (n = 6 for each time point in both groups of injured animals) were again anesthetized and then immediately sacrificed. Sham-operated animals were sacrificed 120 h after the initial anaesthesia (n = 6) and used as controls. For immunohistochemistry, mTBI (n = 12) and sTBI (n = 12) rats were sacrificed at 24 h (n = 6 for both groups) and 120 h (n = 6 for both groups) post injury. A new group of sham-operated animals (n = 6), sacrificed 120 h after the initial anaesthesia, was used as the control group for the immunohistochemical study.

### Gene expression

Total RNA was extracted by homogenizing one hemisphere in Trizol (Invitrogen Life Technologies), using the Ultra-Turrax homogenizer (Janke & Kunkel, Staufen, Ge) at 24,000 rpm/min, to produce a final 10% homogenate (weight:volume). Transcription to cDNA of RNA of samples stored at −80 °C was performed as previously described^56–58^. Subsequent real time-quantitative polymerase chain reaction (RT-qPCR) was carried out using primers designed with the 0.2 version of the Primer3 Input software developed by the Whitehead Institute for Biomedical Research (Cambridge, MA) using as templates the sequences of *Rattus norvegicus* published by the National Center for Biotechnology Information (the list of primers is reported in the Supplementary Table 4). For accurate gene expression measurements with RT-qPCR, results were normalized to the housekeeping gene of β-2-microglobulin (B2M, NM_012512) of *R*. *norvegicus*, selected from twelve candidate reference genes using the geNorm Housekeeping Gene Selection Kit (Primer Design Ltd.). Changes in transcript abundance of tested genes were calculated using the 2^−ΔΔCT^ method described by Livak and Schmittgen^66^.

### Protein expression

Crude homogenates suitable for immunoblot analysis were obtained by homogenizing one brain hemisphere in 15 mM KCl + 1 mM KH_2_PO_4_, pH 7.4, at 24,000 rpm/min for 90 sec in the cold, followed by centrifugation at 18,690 × g for 15 min at 4 °C. Proteins from homogenates stored at −80 °C (10 μg) were separated by 8% SDS–PAGE and transferred onto a nitrocellulose membrane (Bio-Rad, Hertfordshire, UK).

The milk-blocked membrane was then incubated overnight at 4 °C with the anti-CS (1:2000), or anti-OPA1 (1:2000), or anti-OMA1 (1:1000), or anti-DRP1 (1:1000) antibodies (Abcam, UK), subjected to a horseradish-peroxidase–conjugated anti-rabbit IgG (1:5000; Roche Diagnostics, Mannheim, Germany), and revealed with an enhanced chemiluminescence (ECL) Western blot analysis kit (Applied Biosystems) or by the alkaline phosphatase technique. ECL films were scanned densitometrically, and the optical density of bands was quantified using Image J version 1.38 software. Values obtained from the immunoblot quantification of CS were either used to obtain a measure of mitochondrial mass^37, 38^ or to calculate the semi-quantitative measure of the different proteins relative to CS.

### Tissue preparation for immunohistochemistry

Rats were killed by exposure to increasing concentrations of CO_2_ and transcardially perfused with 100 mL of PBS to wash out blood before further perfusion with 100 mL 4% paraformaldehyde (PFA) in PBS at pH 7.4. Dissected brains were post fixed by immersion in 4% PFA in PBS for 2 hours at 4 °C. Cryoprotection was obtained by immersing the brain in PBS enriched with increasing sucrose (10%, 20%, or 30%) for 24 hours at 4 °C, then embedded in optimal cutting temperature (OCT) embedding medium (Thermo Shandon, Runcorn, UK) in peel-away mould containers (Agar Scientific, Essex, UK). Brain immersed in OCT were rapidly frozen in crushed dry ice before storage at −80 °C and later sectioned in the coronal plane at −22 °C using a Bright cryostat microtome (Bright, Huntingdon, UK) at a thickness of 30 µm. Sections were floated in 1 ml/well of cryoprotectant consisting of 50% 0.05 M sodium phosphate buffer, 30% ethylene glycol and 20% glycerol and stored −20 °C.

Floating sections were washed 3 × 5 minutes in PBS containing 0.3% Tween-20 (PBS-T) (Sigma) for permeabilisation before blocking nonspecific antibody binding sites for 20 minutes with 0.5% BSA, 0.3% Tween-20 (all from Sigma), and 15% normal goat serum (Vector Laboratories) in PBS. Sections were then incubated overnight at 4 °C in anti-OPA1 primary antibody (Abcam; UK) followed by 3 washings in PBS-T of 5 minutes each and incubated for 1 hour at RT with Alexa 488 goat-anti rabbit secondary antibody. Sections were then washed 3 × 5 minutes in PBS-T and mounted in Vectorshield mounting medium containing DAPI (Vector Laboratories). Tissue sections from brains of control sham-operated rats incubated with secondary antibody alone were all negatively stained (not shown).

After immunofluorescence staining, sections were viewed on a Zeiss Axioplan 2 epi-fluorescent microscope (Carl Zeiss Ltd.) and images captured using the same exposure times (600 ms) using a Zeiss AxioCam HRc. Immunohistochemistry staining was quantified according to the methods previously described^67^. Briefly, the region of interest used for quantitation of positive staining was defined by a quadrant of the same prescribed size for all brains/injury groups from the same brain region. OPA1 staining was quantified within this defined quadrant of the section and the percentage of immunofluorescent pixels above a standardized background threshold was calculated using ImageJ software (http://imagej.nih.gov/ij/; provided in the public domain by the National Institutes of Health, Bethesda, MD, USA). The threshold level of brightness in the area of the brain was set using intact uninjured brain sections from control animals to define the reference level for injury group analysis of pixel intensity. Images were assigned randomized numbers to ensure blinding of treatment groups during quantification by the assessor.

### Statistical analysis

Statistical analysis was performed by using the Statistical Package for Social Science (SPSS), release 15.0. Normal data distribution was tested using the Kolmogorov–Smirnov test. The within-group comparison at each time was performed by the one-way analysis of variance (ANOVA). Differences across groups were estimated by the two-way ANOVA for repeated measures. Fisher’s protected least square was used as the post hoc test. Only two-tailed p-values of less than 0.05 were considered statistically significant.

## Electronic supplementary material

Tables and Figure
