# Supplementary material for: Fusion or Fission: The Destiny of Mitochondria In Traumatic Brain Injury of Different Severities
Source: Sci Rep. 2017 Aug 23;7:9189. doi: 10.1038/s41598-017-09587-2 (PMC5569027; doi:10.1038/s41598-017-09587-2)
Supplement: Supplementary file 1 — Tables and Figure [file 41598_2017_9587_MOESM1_ESM.doc]

**SUPPLEMENTARY INFORMATION**

**FUSION OR FISSION: THE DESTINY OF MITOCHONDRIA**

**IN TRAUMATIC BRAIN INJURY OF DIFFERENT SEVERITIES**

Valentina Di Pietro^a,b§^, Giacomo Lazzarino^c§^, Angela Maria Amorini^c^, Stefano Signoretti^d^, Lisa J Hill^a,b^, Edoardo Porto^a^, Barbara Tavazzi^c§*^, Giuseppe Lazzarino^e§*^, Antonio Belli^a,b^

^a^Neurotrauma and Ophthalmology Research Group, Institute of Inflammation and Ageing, School of Clinical and Experimental Medicine, College of Medical and Dental Sciences, University of Birmingham, Edgbaston B15 2TT, Birmingham, UK.

^b^National Institute for Health Research Surgical Reconstruction and Microbiology Research Centre, Queen Elizabeth Hospital, Edgbaston B15 2TH, Birmingham, UK.

^c^Institute of Biochemistry and Clinical Biochemistry, Catholic University of Rome, Largo F. Vito 1, 00168 Rome, Italy.

^d^Division of Neurosurgery, Department of Neurosciences Head and Neck Surgery, S. Camillo Hospital, Circonvallazione Gianicolense 87, 00152 Rome, Italy.

^e^Department of Biomedical and Biotechnological Sciences, Division of Medical Biochemistry, University of Catania, Viale A. Doria 6, 95125 Catania, Italy.

^§^These Authors equally contributed to this work

***Correspondence should be addressed to:** Prof. Barbara Tavazzi

Institute of Biochemistry and Clinical Biochemistry, Catholic University of Rome, Largo F. Vito 1, 00168 Rome, Italy; Email: barbara.tavazzi@unicatt.it; phone: +39-0630155135

**or to:** Prof. Giuseppe Lazzarino

Department of Biomedical and Biotechnological Sciences, Division of Medical Biochemistry, University of Catania, Viale A. Doria 6, 95125 Catania, Italy; Email: [lazzarig@unict.it](mailto:lazzarig@unict.it); phone: +39-0957384095


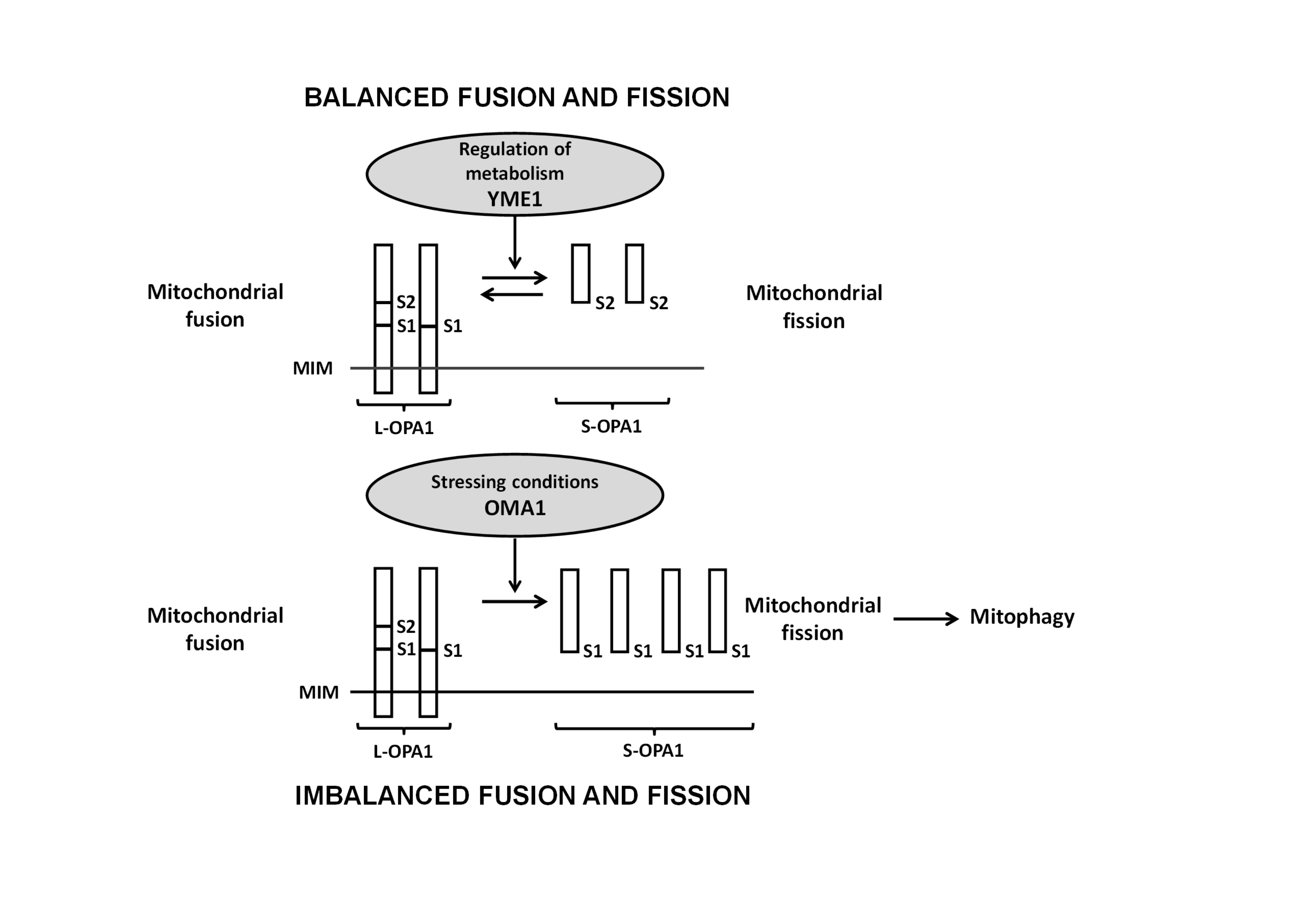


**Figure S1.** Intervention of the proteases cleaving OPA1 and generating differential amounts of L-OPA1 and S-OPA1. Under physiologic conditions of balanced fusion and fission, proteolysis of OPA1 is mainly catalyzed by YME1 at S1 site and producing almost equal amounts of L-OPA1 and S-OPA1, involved in the control of energy metabolism. Under sustained stressing conditions with protracted mitochondrial dysfunction, cleavage is primarily catalyzed by OMA1 at S2 site and forming more S-OPA1 than L-OPA1. Decrease in L-OPA1/S-OPA1 ratio promotes fission and subsequent removal of dysfunctional mitochondria through mitophagy. MIM = mitochondrial inner membrane.

**Table 1.** Summary of the key proteins, and of their respective roles, involved in the regulation of the processes of mitochondrial fusion, fission and mitophagy representing the MQC system.

| **PROTEIN** | **FUNCTION** | **REFERENCE** |
| --- | --- | --- |
| **OPA1** | FUSION of mitochondrial inner membranes and cristae organization and regulator of oxidative phosphorylation. Its proteolytic processing generates L-OPA1 and S-OPA1. Excess of S-OPA1 is pro-apoptotic. | [Ishihara N](http://www.ncbi.nlm.nih.gov/pubmed/?term=Ishihara N%5BAuthor%5D&cauthor=true&cauthor_uid=16778770), *et al.* (2006)  [Song Z](https://www.ncbi.nlm.nih.gov/pubmed/?term=Song Z%5BAuthor%5D&cauthor=true&cauthor_uid=19477917), *et al.* (2007)  [Song Z](https://www.ncbi.nlm.nih.gov/pubmed/?term=Song Z%5BAuthor%5D&cauthor=true&cauthor_uid=19477917), *et al.* (2009)  Alavi MV, Fuhrmann N. (2013) |
| **MFN1** | FUSION of mitochondrial outer membranes. | [Song Z](https://www.ncbi.nlm.nih.gov/pubmed/?term=Song Z%5BAuthor%5D&cauthor=true&cauthor_uid=19477917), *et al.* (2009)  Alavi MV, Fuhrmann N. (2013) |
| **MFN2** | FUSION of mitochondrial inner membranes. | [Song Z](https://www.ncbi.nlm.nih.gov/pubmed/?term=Song Z%5BAuthor%5D&cauthor=true&cauthor_uid=19477917), *et al.* (2009)  Alavi MV, Fuhrmann N. (2013) |
| **DRP1** | FISSION mediator. | Van der Bliek AM, *et al.* (2013)  Alavi MV, Fuhrmann N. (2013) |
| **FIS1** | FISSION mediator. | Van der Bliek AM, *et al.* (2013)  Alavi MV, Fuhrmann N. (2013) |
| **YME1** | PROTEASE directly involved in OPA1 processing: cleavage at S2. Required for balancing the L-OPA1/S-OPA1 ratio in mitochondrial fusion. | [Tondera D](http://www.ncbi.nlm.nih.gov/pubmed/?term=Tondera D%5BAuthor%5D&cauthor=true&cauthor_uid=19360003), *et al.* (2009)  [Duvezin-Caubet S](http://www.ncbi.nlm.nih.gov/pubmed/?term=Duvezin-Caubet S%5BAuthor%5D&cauthor=true&cauthor_uid=17003040), *et al.* (2006)  [Ehses S](http://www.ncbi.nlm.nih.gov/pubmed/?term=Ehses S%5BAuthor%5D&cauthor=true&cauthor_uid=20038678), *et al.* (2009) |
| **OMA1** | PROTEASE directly involved in OPA1 processing: cleavage at S1. Convert L-OPA1 into S-OPA1. Increase in S-OPA1 induces apoptosis. | Alavi MV, Fuhrmann N. (2013)  [Ehses S](http://www.ncbi.nlm.nih.gov/pubmed/?term=Ehses S%5BAuthor%5D&cauthor=true&cauthor_uid=20038678), *et al.* (2009) |
| **PARL** | PROTEASE indirectly involved in OPA1 oligomerization. Catalyzes PINK1 proteolytic cleavage. | Pellegrini L, Scorrano L. (2007)  Sanjuán Szklarz L., Scorrano L. (2012)  Vogt A., *et al.* (2016) |
| **PINK1** | MITOPHAGY activator working upstream PARK2 in the PINK1/PARK2 pathway. Mitochondrial kinase Independently on PARK2, PINK1 regulates complex I and calcium homeostasis. | Alavi MV, Fuhrmann N. (2013)  [Jiang X](https://www.ncbi.nlm.nih.gov/pubmed/?term=Jiang X%5BAuthor%5D&cauthor=true&cauthor_uid=25275009), *et al.* (2014)  Vogt A., *et al.* (2016) |
| **PARK2** | MITOPHAGY activator working downstream PARK2 in the PINK1/PARK2 pathway. | Alavi, M. V., & Fuhrmann, N. (2013)  [Jiang, X](https://www.ncbi.nlm.nih.gov/pubmed/?term=Jiang X%5BAuthor%5D&cauthor=true&cauthor_uid=25275009)., *et al.* (2014)  Vogt A., *et al.* (2016) |

**Table 2.** Summary of the time course changes of the expressions of the gene regulating the synthesis of the proteins controlling the MQC system. The specific role of each protein product is schematically illustrated in Table 1 and Figure 1 of the Supplementary information.

|  | **Time post injury (hours)** |  | **OPA1** | **MFN1** | **MFN2** | **YME1** | **OMA1** | **PARL** | **DRP1** | **FIS1** | **PINK1** | **PARK2** |
| --- | --- | --- | --- | --- | --- | --- | --- | --- | --- | --- | --- | --- |
|  |  |  |  |  |  |  |  |  |  |  |  |  |
|  | **6** |  | 1.19 | 1.68 (↑) | 1.12 | 1.58 | 0.81 (↓) | 1.40 (↑) | 0.67 (↓) | 1.17 | 0.61 (↓) | 1.12 |
| **mTBI** | **24** |  | 1.40 (↑) | 1.52 (↑) | 1.06 | 1.63 | 0.70 (↓) | 0.88 | 0.44 (↓) | 1.16 | 1.09 | 1.22 |
|  | **48** |  | 2.02 (↑) | 1.43 (↑) | 2.58 (↑) | 2.92 (↑) | 0.72 (↓) | 1.92 (↑) | 0.45 (↓) | 1.30 (↑) | 1.13 | 0.81 (↓) |
|  | **120** |  | 3.61 (↑) | 1.77 (↑) | 3.55 (↑) | 3.46 (↑) | 1.01 | 2.50 (↑) | 0.45 (↓) | 1.16 | 1.90 (↑) | 0.65 (↓) |
|  |  |  |  |  |  |  |  |  |  |  |  |  |
|  | **6** |  | 1.48 (↑) | 0.47 | 0.66 | 1.41 | 1.70 (↑) | 0.97 | 1.50 (↑) | 1.85 (↑) | 0.90 | 0.96 |
| **sTBI** | **24** |  | 1.00 | 0.34 | 0.62 | 1.52 | 1.47 (↑) | 0.72 | 1.30 | 1.73 (↑) | 1.01 | 1.15 |
|  | **48** |  | 1.29 | 0.42 | 1.17 | 2.15 (↑) | 1.78 (↑) | 1.11 | 1.55 (↑) | 2.34 (↑) | 1.51 (↑) | 1.67 (↑) |
|  | **120** |  | 1.66 (↑) | 0.31 | 1.14 | 1.67 | 1.58 (↑) | 1.09 | 1.34 | 2.09 (↑) | 1.56 (↑) | 2.12 (↑) |

Values are those indicated in Figures 1, 2, 3 and 4, and are reported in the Table as fold change respect to the corresponding value of controls. Standard deviations have been omitted for the sake of clarity.

Arrows indicate significant upregulation (↑) or downregulation (↓) of the gene expression compared to controls (p < 0.05).

**Table 3.** Summary of the time course changes of the expressions of selected proteins involved in the regulation of the MQC system. The specific role of each protein is schematically illustrated in Table 1 and Figure 1 of the Supplementary Materials.

|  | **Time post injury (hours)** | **CS** | **L-OPA** | **S-OPA** | **L-OPA/S-OPA** | **OMA1** | **DRP1** |
| --- | --- | --- | --- | --- | --- | --- | --- |
|  |  |  |  |  |  |  |  |
|  | **6** | 1.14 | 0.96 | 0.68 ↓ | 1.37 | 0.85 | 0.62 ↓ |
| **mTBI** | **24** | 0.94 | 1.26 | 0.40 ↓ | 3.08 | 0.68 ↓ | 0.53 ↓ |
|  | **48** | 1.48 ↑ | 0.91 | 0.69 ↓ | 1.30 | 0.72 | 0.54 ↓ |
|  | **120** | 1.49 ↑ | 1.40 ↑ | 0.88 | 1.60 | 0.91 | 0.47 ↓ |
|  |  |  |  |  |  |  |  |
|  | **6** | 0.73 ↓ | 0.30 ↓ | 0.98 | 0.30 ↓ | 1.55 ↑ | 0.94 |
| **sTBI** | **24** | 0.67 ↓ | 0.36 ↓ | 0.98 | 0.36 ↓ | 1.28 | 1.22 ↑ |
|  | **48** | 0.80 ↓ | 0.23 ↓ | 0.93 | 0.25 ↓ | 1.19 | 1.07 |
|  | **20** | 0.82 ↓ | 0.26 ↓ | 0.90 | 0.26 ↓ | 1.73 ↑ | 1.33 ↑ |

Values are those indicated in Figures 7, 8, and 9, and are reported in the Table as fold change respect to the corresponding value of controls. Standard deviations have been omitted for the sake of clarity.

Arrows indicate significant upregulation (↑) or downregulation (↓) of the protein expression (p < 0.05 compared to controls).

**Table 4.** Sequences of primers used to evaluate the expressions of the genes regulating the synthesis of the main proteins involved in mitochondrial fusion, fission and mitophagy.

| **Gene name** | **Accession Number** | **Forward** | **Reverse** |
| --- | --- | --- | --- |
| OPA1 | [NM_133585.3](http://www.ncbi.nlm.nih.gov/nuccore/NM_133585.3) | CAGCTGGCAGAAGATCTCAAG | CATGAGCAGGATTTTGACACC |
| MFN1 | [NM_138976.1](http://www.ncbi.nlm.nih.gov/nuccore/NM_138976.1) | CGGAGGCATATGAAAGTGGC | CCATCAGTTCCCTCCACACT |
| MFN2 | [NM_130894.4](http://www.ncbi.nlm.nih.gov/nuccore/NM_130894.4) | TTGACTCCAGCCATGTCCAT | GGTGACGATGGAGTTGCATC |
| YME1 | [NM_053682.2](http://www.ncbi.nlm.nih.gov/nuccore/NM_053682.2) | AGGATGCAATGCCAATCAAT | TGCCACTCTTCCTCCCATAC |
| DRP1 | [NM_053655.3](http://www.ncbi.nlm.nih.gov/nuccore/NM_053655.3) | TGGAGATGGTGGTCAGGAAC | TTTCGTGCAACTGGAACTGG |
| FIS1 | [NM_001105919.1](http://www.ncbi.nlm.nih.gov/nuccore/NM_001105919.1) | ATGGATGCCCAGAGATGAAG | ACGATGCCTCTACGGATGTC |
| OMA1 | [NM_001106669.1](http://www.ncbi.nlm.nih.gov/nuccore/NM_001106669.1) | ACCAGTGCAAAAGCTCCTTG | TGTTCCTCTTCACGCTGTCT |
| PARL | [NM_001035249.1](http://www.ncbi.nlm.nih.gov/nuccore/NM_001035249.1) | AGGCTGTGCATTTGGTTCAG | TTTCCTTCCTTTTGTGGCCG |
| PINK1 | [NM_001106694.1](http://www.ncbi.nlm.nih.gov/nuccore/NM_001106694.1) | CCAAACACCTTGGCCTTCTA | CTTAAGATGGCTTCGCTGGA |
| PARK2 | [NM_020093.1](http://www.ncbi.nlm.nih.gov/nuccore/NM_020093.1) | ACCCACCTACCACAGCTTTT | CAAGGTGAGGGTTGCTTGTC |
